# Global Deletion of 11β-HSD1 Prevents Muscle Wasting Associated with Glucocorticoid Therapy in Polyarthritis

**DOI:** 10.3390/ijms22157828

**Published:** 2021-07-22

**Authors:** Justine M. Webster, Michael S. Sagmeister, Chloe G. Fenton, Alex P. Seabright, Yu-Chiang Lai, Simon W. Jones, Andrew Filer, Mark S. Cooper, Gareth G. Lavery, Karim Raza, Ramon Langen, Rowan S. Hardy

**Affiliations:** 1 Institute for Metabolism and Systems Research, College of Medical and Dental Sciences, University of Birmingham, Birmingham B15 2TT, UK; M.Sagmeister@bham.ac.uk (M.S.S.); chloe.fenton@uni-wuerzburg.de (C.G.F.); G.G.Lavery@bham.ac.uk (G.G.L.); r.hardy@bham.ac.uk (R.S.H.); 2 Department of Respiratory Medicine, NUTRIM School of Nutrition and Translational Research in Metabolism, Faculty of Health, Medicine and Life Sciences, Maastricht University, 6211 LK Maastricht, The Netherlands; r.langen@maastrichtuniversity.nl; 3 Research into Inflammatory Arthritis Centre Versus Arthritis, Institute of Inflammation and Ageing, University of Birmingham, Birmingham B15 2TT, UK; A.FILER@bham.ac.uk (A.F.); K.Raza@bham.ac.uk (K.R.); 4 School of Sport, Exercise and Rehabilitation Sciences, University of Birmingham, Birmingham B15 2TT, UK; AXS564@student.bham.ac.uk (A.P.S.); Y.Lai.1@bham.ac.uk (Y.-C.L.); 5 MRC Arthritis Research UK Centre for Musculoskeletal Ageing Research, University of Birmingham, Birmingham B15 2TT, UK; S.W.Jones@bham.ac.uk; 6 ANZAC Research Institute, The University of Sydney, Sydney, NSW 2139, Australia; mark.cooper@sydney.edu.au; 7 Department of Rheumatology, Sandwell and West Birmingham NHS Trust, Birmingham B71 4HJ, UK; 8 Institute of Clinical Science, University of Birmingham, Birmingham B15 2TT, UK

**Keywords:** sarcopenia, myopathy, steroids, adverse effects, 11beta hydroxysteroid dehydrogenase type 1, rheumatoid arthritis, inflammation

## Abstract

Glucocorticoids provide indispensable anti-inflammatory therapies. However, metabolic adverse effects including muscle wasting restrict their use. The enzyme 11beta-hydroxysteroid dehydrogenase type 1 (11β-HSD1) modulates peripheral glucocorticoid responses through pre-receptor metabolism. This study investigates how 11β-HSD1 influences skeletal muscle responses to glucocorticoid therapy for chronic inflammation. We assessed human skeletal muscle biopsies from patients with rheumatoid arthritis and osteoarthritis for 11β-HSD1 activity ex vivo. Using the TNF-α-transgenic mouse model (TNF-tg) of chronic inflammation, we examined the effects of corticosterone treatment and 11β-HSD1 global knock-out (11βKO) on skeletal muscle, measuring anti-inflammatory gene expression, muscle weights, fiber size distribution, and catabolic pathways. Muscle 11β-HSD1 activity was elevated in patients with rheumatoid arthritis and correlated with inflammation markers. In murine skeletal muscle, glucocorticoid administration suppressed *IL6* expression in TNF-tg mice but not in TNF-tg^11βKO^ mice. TNF-tg mice exhibited reductions in muscle weight and fiber size with glucocorticoid therapy. In contrast, TNF-tg^11βKO^ mice were protected against glucocorticoid-induced muscle atrophy. Glucocorticoid-mediated activation of catabolic mediators (*FoxO1*, *Trim63*) was also diminished in TNF-tg^11βKO^ compared to TNF-tg mice. In summary, 11β-HSD1 knock-out prevents muscle atrophy associated with glucocorticoid therapy in a model of chronic inflammation. Targeting 11β-HSD1 may offer a strategy to refine the safety of glucocorticoids.

## 1. Introduction

Glucocorticoids are indispensable in modern medicine as highly effective anti-inflammatory medications. They are widely used with applications ranging from autoimmune diseases to cancer, organ transplantation, or COVID-19 pneumonia. Their capacity to control inflammation relies on direct action on immune cells [1,2]. However, action on tissues besides the immune system leads to a characteristic pattern of adverse effects that restricts their long-term use [2,3]. In skeletal muscle, glucocorticoids cause weakness and atrophy through pathways including reduced anabolic insulin-like growth factor 1 (IGF1)/insulin signaling and enhanced catabolic signaling via the ubiquitin-proteosome system [4]. Patients suffer detrimental consequences from muscle wasting in the form of reduced mobility, higher falls and fracture risk, higher hospitalization rates and increased mortality [5].

The enzyme 11beta-hydroxysteroid dehydrogenase type 1 (11β-HSD1) modulates the signal strength of glucocorticoids within target tissues, including in skeletal muscle [6]. It converts inactive glucocorticoids (cortisone or prednisone in humans; dehydrocorticosterone (DHC) in rodents) to active glucocorticoids (cortisol/prednisolone/corticosterone), amplifying the local glucocorticoid signal. 11β-HSD1 has been implicated in a range of metabolic pathologies [6]. In particular, suppression of 11β-HSD1 protects against muscle wasting from glucocorticoid excess, highlighting 11β-HSD1 as a potential target for therapeutic intervention [7,8]. Pharmaceutical inhibitors of 11β-HSD1 have been developed and are in clinical trials for diabetes and other metabolic diseases [9,10,11].

Inflammation enhances the activity of 11β-HSD1. Inflammatory cytokines such as TNF-α upregulate 11β-HSD1 expression, providing a local negative feedback to control inflammation with local anti-inflammatory glucocorticoid activation [12,13]. Inflammation also acts systemically to augment circulating glucocorticoids (activation of the hypothalamic-pituitary-adrenal (HPA) axis), as well as acting directly on skeletal muscle to cause myopathy [14]. Hence, compound effects on skeletal muscle depend on complex interactions between inflammation, 11β-HSD1 activity and glucocorticoid signaling.

Our previous work showed that both the genetic deletion of 11β-HSD1 and exogenous glucocorticoid, administered at a dose sufficient to suppress disease activity, exacerbate the muscle wasting phenotype in a model of polyarthritis with chronic inflammation [13,15]. This study addresses whether suppression of 11β-HSD1 function will protect or harm skeletal muscle metabolism when chronic inflammation and exogenous glucocorticoids are combined in a murine model of glucocorticoid-treated polyarthritis. Our data show that global 11β-HSD1 knock out (11βKO) has a protective effect against skeletal muscle wasting when mice with chronic TNF-α overexpression receive oral corticosterone. This offers new insights into the driving factors for muscle wasting in glucocorticoid-treated inflammatory disease, with implications for strategies to mitigate the adverse effects of glucocorticoid treatments in the future.

## 2. Results

### 2.1. Cortisol Activation by 11β-HSD1 Is Increased in Skeletal Muscle in Rheumatoid Arthritis and Correlates with Inflammation

**Figure 1:**
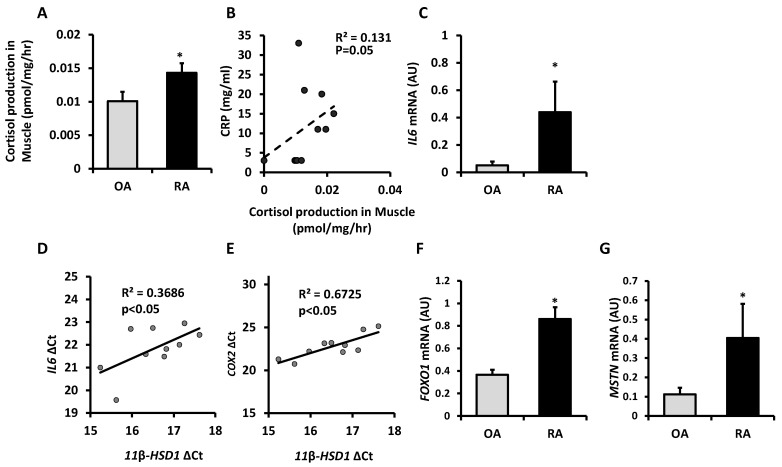
(**A**) Glucocorticoid activation by 11β-HSD1 in ex vivo muscle explants freshly isolated after joint replacement surgery from patients with rheumatoid arthritis (RA, n = 10) and osteoarthritis (OA n = 12) determined by scanning thin-layer chromatography. (**B**) A significant correlation was seen between glucocorticoid activation (oxoreductase activity) and the serum C-reactive protein (CRP) measured before surgery in RA patients. (**C**) Gene expression (AU) of *IL6* determined by RT qPCR in muscle homogenates from RA (n = 6) and OA (n = 4) patients. (**D**) Correlation between gene expression (ΔCt) of 11β-HSD1 with *IL6* and (**E**) *COX2* determined by RT qPCR in muscle homogenates from RA (n = 6) and OA (n = 4) patients. (**F**) Gene expression (AU) of *FOXO1* and (**G**) *MSTN* determined by RT qPCR in muscle homogenates from RA (n = 5) and OA (n = 4) patients. Values are expressed as mean ± standard error. Statistical significance was determined using an unpaired *t*-test. * *p* < 0.05.

Inflammatory upregulation of 11β-HSD1 has previously been demonstrated when skeletal muscle samples from patients with osteoarthritis (OA) were stimulated with TNF-α ex vivo [13]. Here, we investigated whether inflammatory upregulation of 11β-HSD1 is evident in muscle biopsies from patients with rheumatoid arthritis (RA), a condition with chronic systemic inflammation. Patients with OA and RA had similar age, but systemic inflammation markers were higher in patients with RA including C-reactive protein (CRP) and erythrocyte sediment rate (ESR) (Table 1). Exclusion criteria stipulated that no patient received current oral glucocorticoid therapy. Measuring ex vivo cortisone to cortisol conversion rate, we confirmed that 11β-HSD1 enzymatic activity was higher in muscle biopsies taken from patients with RA compared to patients with OA (mean ± std. error: 0.014 ± 0.001 pmol/mg tissue/h vs. 0.010 ± 0.001 pmol/mg tissue/h respectively, *p* < 0.05; Figure 1A). Among patients with RA, muscle 11β-HSD1 activity correlated with circulating CRP levels (R^2^ = 0.131, *p* = 0.05; Figure 1B). Real-time PCR showed higher expression of the inflammatory cytokine *IL6* in muscle samples from patients with RA compared to patients with OA (fold change = 8.65, *p* < 0.05; Figure 1C). Expression of this tissue-specific marker of inflammation correlated with expression of 11β-HSD1 among patients with RA (R^2^ = 0.369, *p* < 0.05; Figure 1D). Similarly, expression of the inducible inflammatory activator *COX2* correlated with 11β-HSD1 expression in muscle biopsies from patients with RA (R^2^ = 0.672, *p* < 0.05; Figure 1E). Finally, muscle samples from patients with RA compared to OA exhibited higher expression of Forkhead box protein O1 (*FOXO1*) and myostatin (*MSTN*), negative regulators of muscle growth (fold changes 2.35, *p* < 0.05 and 3.62, *p* < 0.05 respectively; Figure 1F,G). Taken together, these data demonstrate that 11β-HSD1 activity is elevated in the skeletal muscle of patients with RA relative to patients with OA, and that 11β-HSD1 upregulation correlates with systemic and tissue-specific markers of inflammation.

### 2.2. Deletion of 11β-HSD1 in a Model of Chronic Inflammation Causes Resistance to the Anti-Inflammatory Responses of Therapeutic Glucocorticoid in Muscle

**Figure 2:**
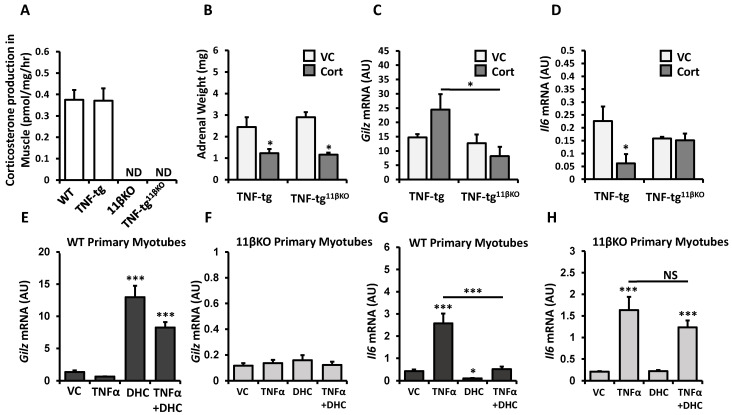
(**A**) Corticosterone production (pmol/mg/hour) determined by scanning thin layer chromatography in ex vivo tibialis anterior muscle biopsy isolated from wild type (WT), TNF-transgenic (TNF-tg), 11β-HSD1 knock out (11βKO) and TNF-tg^11βKO^ animals. (**B**) Adrenal weights and gene expression (AU) of (**C**) *Gilz* and (**D**) *Il6* determined by RT qPCR in muscle homogenates from TNF-tg and TNF-tg^11βKO^ animals receiving either vehicle or corticosterone (100 µg/mL) in the drinking water for 3 weeks. Gene expression (AU) of (**E**,**F**) *Gilz* and (**G**,**H**) *Il6* in primary murine muscle cultures from WT or single mutant 11βKO animals treated with recombinant TNFα and/or dehydrocorticosterone (DHC) determined by RT qPCR. Values are expressed as mean ± SE, n = 6 per group for animal experiments and n = 3 per group for primary culture. Statistical significance was determined using two-way analysis of variance with Tukey post hoc analysis. * *p* < 0.05, *** *p* < 0.001. NS, not significant; WT, Wild type; 11βKO, 11β-HSD1 genetic deletion; VC, Vehicle Control; Cort, Corticosterone; DHC, Dehydrocorticosterone; ND, not detectable.

11β-HSD1 function has emerged as essential for glucocorticoid therapy to suppress joint inflammation in models of polyarthritis [16]. We therefore investigated how deletion of 11β-HSD1 affects anti-inflammatory responses in muscles when mice with inflammation from transgenic human TNF-α overexpression are treated with oral corticosterone. The skeletal muscle tissue from 11βKO mice exhibited no detectable activity to activate DHC to corticosterone (Figure 2A). Systemic treatment with corticosterone led to a 50% adrenal weight reduction in TNF-tg mice relative to untreated controls (*p* < 0.05; Figure 2B). For TNF-tg^11βKO^ mice, adrenal weight reduction with corticosterone treatment was 60% relative to untreated counterparts (*p* < 0.05; Figure 2B). Oral corticosterone treatment in TNF-tg mice led to a trend for higher muscle mRNA expression of *Gilz*, a glucocorticoid responsive gene with anti-inflammatory properties (Figure 2C). However, such a trend for corticosterone-induced *Gilz* expression was not apparent in TNF-tg^11βKO^ mice. In direct comparison of corticosterone-treated groups, muscle *Gilz* expression was significantly higher in TNF-tg mice than in TNF-tg^11βKO^ mice (fold change = 2.98, *p* < 0.05). Examination of *Il6* mRNA expression in muscle yielded consistent results. While corticosterone administration suppressed expression of pro-inflammatory *Il6* in TNF-tg mice (fold change = 0.27, *p* < 0.05; Figure 2D), corticosterone was rendered ineffective in TNF-tg^11βKO^ mice (fold change = 0.96, *p*-value NS). Primary muscle cultures of WT and 11βKO animals confirmed that 11βKO muscle cells are unresponsive to the inactive glucocorticoid DHC and its immunosuppressive effects. DHC does not activate the glucocorticoid receptor directly but relies on conversion to active corticosterone by 11β-HSD1 to trigger glucocorticoid responses. Accordingly, DHC stimulation markedly enhances *Gilz* mRNA expression in WT muscle cells, but not in 11βKO cells (Figure 2E,F). Similarly, DHC stimulation markedly suppresses TNF-induced *Il6* mRNA expression in WT muscle cells, but not in 11βKO cells (Figure 2G,H). Together, these data reveal that anti-inflammatory responses of glucocorticoids in skeletal muscle are dependent on 11β-HSD1.

### 2.3. 11β-HSD1 Mediates Muscle Wasting in Response to Therapeutic Glucocorticoids in Chronic Inflammation

Global deletion of 11β-HSD1 has previously been shown to exacerbate myopathy in the inflammatory model of TNF-tg mice but protect against myopathy in the glucocorticoid excess model of WT mice receiving oral corticosterone [13,15]. Next, we explored the role of 11β-HSD1 for muscle atrophy in a combined model of TNF-tg mice treated with corticosterone.

**Figure 3:**
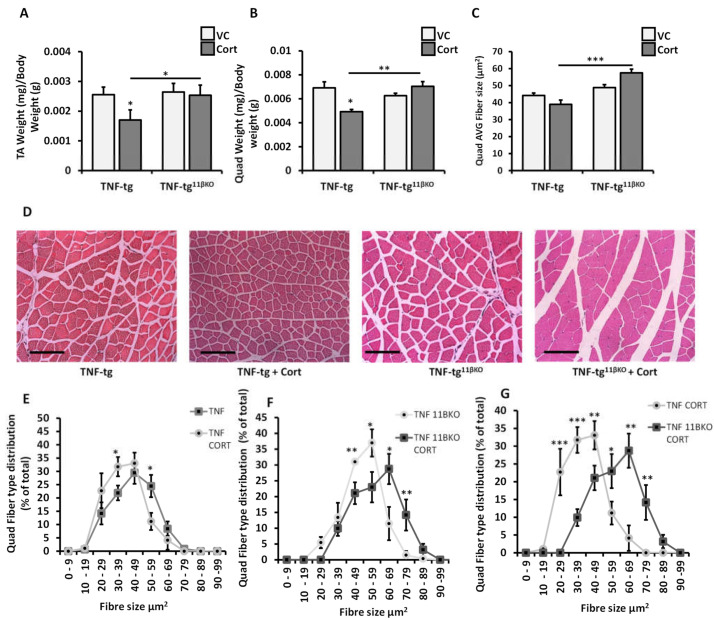
Total (**A**) Tibialis anterior (TA) and (**B**) quadriceps muscle weights relative to total bodyweight and (**C**) average quadriceps muscle fibre cross sectional area (μm^2^) in TNF-tg, and TNF-tg^11βKO^ animals receiving either vehicle or corticosterone (100 µg/mL) in the drinking water for 3 weeks. (**D**) Representative images of quadriceps muscle sections and (**E**–**G**) distribution of quadriceps muscle fibre cross-sectional area determined using Image J in paraffin embedded sections in TNF-tg, and TNF-tg^11βKO^ animals receiving either vehicle or corticosterone (100 µg/mL) in the drinking water for three weeks. Values are expressed as mean ± standard error of six animals per group. Statistical significance was determined using two-way ANOVA with a Tukey post hoc analysis. * *p* < 0.05, ** *p* < 0.005, *** *p* < 0.001 (scale bars, 50 μm). VC = Vehicle Control, Cort = Corticosterone.

Tibialis anterior and quadriceps muscle weights (standardized to total body weights) were assessed in the four experimental groups of vehicle-treated TNF-tg mice, corticosterone-treated TNF-tg mice, vehicle-treated TNF-tg^11βKO^ mice and corticosterone-treated TNF-tg^11βKO^ mice (Figure 3A,B). In TNF-tg mice, corticosterone treatment reduced standardized tibialis anterior and quadriceps muscle weights compared to vehicle control (−33%, *p* < 0.05 and −29%, *p* < 0.05 respectively). In TNF-tg^11βKO^ mice however, corticosterone treatment had no significant effect on muscle weights relative to vehicle treatment (TA: −4%, *p*-value NS and quadriceps: +12%, *p*-value NS). In direct comparison of corticosterone-treated TNF-tg animals and corticosterone-treated TNF-tg^11βKO^ animals, standardized muscle weights were significantly greater in the latter group (TA: +49%, *p* < 0.05 and quadriceps: +43%, *p* < 0.01).

Quantitative analysis of average muscle fiber size and distribution yielded matching results. The average fiber cross-sectional area was significantly larger in corticosterone-treated TNF-tg^11βKO^ mice than in treated TNF-tg mice (57.5 µm^2^ vs. 39.0 µm^2^, *p* < 0.001; Figure 3C and Supplementary Figure S2). Examined in closer detail, corticosterone treatment for TNF-tg mice produced a left shift in fiber size distribution (i.e., higher frequency of small fibers and lower frequency of large fibers (Figure 3E)). In contrast, corticosterone treatment for TNF-tg^11βKO^ mice produced a right shift in fiber size distribution (Figure 3F). When muscle histology of corticosterone-treated TNF-tg and TNF-tg^11βKO^ animals is compared directly, lack of 11β-HSD1 function produces a marked right shift in fiber size distribution (i.e., an abundance of larger fibers and scarcity of smaller fibers (Figure 3G)). To summarize the data from muscle weights and histology, suppression of 11β-HSD1 activity eliminates the atrophic effects of glucocorticoid therapy on skeletal muscle in the context of chronic inflammation.

### 2.4. 11β-HSD1 KO Diminishes Activation of Muscle Catabolic Pathways in Response to Therapeutic Glucocorticoid

**Figure 4:**
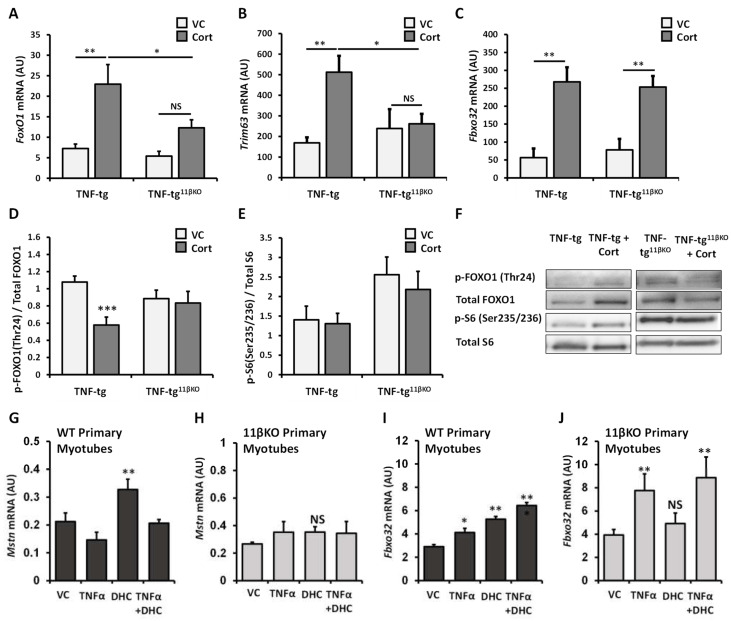
Gene expression (AU) of (**A**) *Foxo1*, (**B**) *Trim63*, and (**C**) *Fbxo32* determined by RT qPCR in muscle homogenates from TNF-tg, and TNF-tg^11βKO^ animals receiving either vehicle or corticosterone (100 µg/mL) in the drinking water for 3 weeks. (**D**) The p-FOXO1(Thr24)/Total FOXO1 and (**E**) p-S6(Ser235/236)/Total S6 ratios and (**F**) representative western blot staining after loading of 20 μg of protein and normalization to Ponceau staining in quadriceps for TNF-tg and TNF-tg^11βKO^ animals receiving either vehicle or corticosterone (100 µg/mL) in the drinking water for 3 weeks. Gene expression (AU) of (**G**,**H**) *Mstn* and (**I**,**J**) *Fbxo32* in primary murine muscle cultures from WT and single mutant 11βKO animals treated with recombinant TNFα and/or dehydrocorticosterone (DHC) determined by RT qPCR. Values are expressed as mean ± SE, n = 6 per group for animal experiments and n = 3 per group for primary culture. Statistical significance was determined using two-way analysis of variance with Tukey post hoc analysis. * *p* < 0.05, ** *p* < 0.005, *** *p* < 0.001. WT, Wild type; 11βKO, 11β-HSD1 genetic deletion; VC, Vehicle Control; Cort, Corticosterone; DHC, Dehydrocorticosterone.

Inflammation and glucocorticoids are known to mediate muscle atrophy through induction of overlapping catabolic signaling pathways. To elucidate the mechanism by which 11β-HSD1 contributes to muscle wasting in our model of glucocorticoid therapy for chronic inflammation, we examined several regulators of muscle protein metabolism. *FoxO1* is a transcription factor and negative regulator of muscle growth [4]. Corticosterone induced *FoxO1* mRNA expression in muscle of TNF-tg mice (fold change = 3.16, *p* < 0.05), but this response was diminished in animals with 11β-HSD1 deletion (fold change = 2.28, *p*-value NS; Figure 4A). Data on phosphorylation of FOXO1, an inactivating modification, mirrors data on total FOXO1 expression. Corticosterone lowered relative phosphorylation/inactivation of FOXO1 in TNF-tg mice (*p* < 0.001), but not in TNF-tg^11βKO^ mice (*p*-value NS; Figure 4D). *Trim63* (also known as *MuRF1*), an E3 ubiquitin ligase involved in proteolysis, was also upregulated by corticosterone treatment in muscle of TNF-tg mice (fold change = 3.03, *p* < 0.01), but not in TNF-tg^11βKO^ mice (fold change = 1.10, *p*-value NS; Figure 4B). However, the same pattern was less clear for *Fbxo32*, a constituent of the catabolic ubiquitin protein ligase complex. mRNA expression of *Fbxo32* in muscle was upregulated by corticosterone in both TNF-tg and TNF-tg^11βKO^ mice (fold changes 4.75, *p* < 0.01 and 3.24, *p* < 0.01 respectively; Figure 4C). Finally, phosphorylation of ribosomal protein S6, a regulator of mRNA translation and protein synthesis, was not significantly different between TNF-tg and TNF-tg^11βKO^ mice and appeared unaffected by corticosterone treatment in either animal group (Figure 4E,F). To gain further insights, we conducted in vitro experiments using primary muscle cell cultures from WT and 11βKO mice. Expression of myostatin (*Mstn*), an inhibitory factor for muscle growth, increased on stimulation with the inactive glucocorticoid DHC in wild type muscle cells, but not in 11βKO muscle cells (Figure 4G,H). TNF-α did not induce *Mstn*. Expression of *Fbxo32* increased with both DHC and TNF-α stimulation with additive effects in wild type muscle cells, but the response to DHC was again absent in 11βKO muscle cells (Figure 4I,J). Summing up the observations on regulators of muscle metabolism, glucocorticoid therapy in our model of chronic inflammation potently activates muscle catabolic signaling pathways. Genetic deletion of 11β-HSD1 diminishes the glucocorticoid-triggered activation of catabolic signals.

## 3. Discussion

This study reports new knowledge on enhanced glucocorticoid activation in skeletal muscle of patients with rheumatoid arthritis. Furthermore, it provides new insights into the role of 11β-HSD1 for exogenous glucocorticoid therapy in an inflammatory disease model, building on previous research that examined the role of 11β-HSD1 either for exogenous glucocorticoid excess in non-inflammatory models or endogenous glucocorticoid effects in inflammatory models [8,13]. In the therapeutic model of TNF-transgenic mice receiving oral corticosterone as anti-inflammatory treatment, genetic deletion of the glucocorticoid activating enzyme 11β-HSD1 abolished immunosuppressive responses in muscle. At the same time, 11β-HSD1 deletion mitigated the adverse effects of glucocorticoid therapy on skeletal muscle, protecting against muscle atrophy and diminishing activation of catabolic signaling pathways.

Examination of human skeletal muscle biopsies corroborates that inflammation upregulates enzymatic activity of 11β-HSD1 to activate glucocorticoids locally within muscle. 11β-HSD1 activity was higher for patients with chronic inflammation from rheumatoid arthritis compared to age-matched patients with osteoarthritis. Furthermore, 11β-HSD1 activity correlated with systemic and tissue-specific markers of inflammation among patients with rheumatoid arthritis. The confirmation of 11β-HSD1 upregulation in skeletal muscle of humans with inflammatory disease concords with previous findings. 11β-HSD1 activity in human muscle rises following ex vivo stimulation with TNF-α [13], or following a major inflammatory insult like abdominal surgery [17]. Furthermore, 11β-HSD1 activity by systemic measures or in tissues besides muscle correlates consistently with inflammation in a range of human observational studies [18,19,20,21]. Cell culture experiments clarified that inflammatory cytokines like TNF-α or IL1β directly enhance 11β-HSD1 expression via the NFκB signaling pathway [12,13]. Altogether, there is strong evidence for upregulation of 11β-HSD1 activity in skeletal muscle by inflammation.

The inflammatory upregulation of 11β-HSD1 in skeletal muscle may provide a physiological defense against excessive inflammatory injury, locally amplifying and targeting immunosuppressive actions of endogenous steroids. Deletion of 11β-HSD1 accordingly exacerbates myopathy in TNF-tg mice with untreated inflammation [13]. Nevertheless, tissue-specific markers of inflammation as well as muscle atrophy markers remained elevated in our cohort with rheumatoid arthritis compared to osteoarthritis. This was despite elevated 11β-HSD1 activity in muscle and in the absence of concurrent glucocorticoid therapy. It remains unclear whether catabolic signaling in this setting of human disease is predominantly due to persisting inflammatory injury or due to augmented atrophic action of endogenous steroids. Future studies with detailed phenotyping following anti-inflammatory or 11β-HSD1 blocking interventions would be needed to address this question.

The significance of 11β-HSD1 function for the anti-inflammatory efficacy of therapeutic glucocorticoids is demonstrated in the TNF-tg animal model. Deletion of 11β-HSD1 led to loss of immunosuppressive responses in skeletal muscle that are normally seen with corticosterone treatment. Morgan et al. previously showed that 11β-HSD1 function is required for glucocorticoid action on muscle in terms of myopathic effects in a model of exogenous glucocorticoid excess [8]. Our data confirm that 11β-HSD1 function is equally important for glucocorticoid action on muscle in terms of immunosuppressive effects in a model of therapeutic glucocorticoid use. These observations in muscle tissue mirror findings from other tissue types. Corticosterone treatment failed to suppress joint inflammation in mouse models of polyarthritis when 11β-HSD1 function was absent [16]. Notably, this is true for administration of active glucocorticoids. Corticosterone treatment led to equivalent adrenal atrophy in TNF-tg and TNG-tg^11βKO^ animals, indicating that glucocorticoid-mediated suppression of the HPA axis was preserved. Considering these findings together suggests that 11β-HSD1 is critical for sustaining or targeting activity of administered glucocorticoids to exert anti-inflammatory effects.

Genetic deletion of 11β-HSD1 protected against muscle wasting in our model of glucocorticoid therapy for inflammatory disease. Results from muscle weights and fiber size distribution were supported by analysis of catabolic signaling pathways. 11βKO diminished activation of *FoxO1* and *Trim63* (also known as *MuRF1*) in muscle of TNF-tg mice receiving corticosterone. *Fbxo32* was not suppressed in this specific context, suggesting active glucocorticoid administration without signal augmentation by 11β-HSD1 was sufficient for this response. The greater glucocorticoid sensitivity of *Fbxo32* may reflect synergistic activation with TNF, as observed in our in vitro data, possibly via the transcription factor C/EBPβ [22,23].

Glucocorticoid-induced muscle atrophy was prevented despite failure of immunosuppression and higher inflammatory markers in muscle from 11β-HSD1-deficient TNF-tg^11βKO^ animals relative to the 11β-HSD1-expressing TNF-tg control group. These results are different from observations from untreated TNF-tg mice. 11βHSD1 deletion had no effect on muscle phenotype of vehicle-treated TNF-tg mice in this study, or exacerbated muscle wasting in previous reports [13]. Apparent discrepancies in this regard may be attributed to the timepoint of muscle phenotype assessment, which was earlier in the current study to pre-empt treatment-resistant phenotype divergence. Matching our results from glucocorticoid-treated TNF-tg mice, 11β-HSD1 deletion also mitigated myopathy in wild-type animals receiving excess exogenous glucocorticoid [8]. Altogether, this supports an interpretation that myopathic effects from glucocorticoid therapy predominated over myopathic effects of inflammation in our model of TNF-tg mice treated with oral corticosterone. Further validation of this concept in human disease is needed. Nevertheless, it matches clinical experiences of muscle wasting as a common adverse effect with glucocorticoid therapy for inflammatory disease [3,24]. In parallel with animal models receiving exogenous glucocorticoids, glucocorticoid doses for immunosuppression in clinical practice are much above physiological equivalents. The principal observations from our study (elevated 11β-HSD1 activity in muscle in the inflammatory disease rheumatoid arthritis and dependency of adverse glucocorticoid effects on 11β-HSD1 function in the animal model) considered together allow the conclusion that inflammatory upregulation of 11β-HSD1 in skeletal muscle may increase patients’ susceptibility to glucocorticoid-mediated myopathy. Therefore, there is a strong argument that 11β-HSD1 activity has a critical contribution to myopathy from glucocorticoid therapy.

Our study highlights a central role of 11β-HSD1 activity for action of glucocorticoids on skeletal muscle, in terms of beneficial anti-inflammatory effects as well as harmful myopathic effects. Using primary muscle cell cultures from wild-type and 11βKO mice without transgenic TNFα expression, we showed that skeletal muscle tissue has sufficient capacity for glucocorticoid activation to downregulate cytokines and upregulate catabolic mediators in an auto-/paracrine manner. Even though minor variations between separate primary cultures are possible, this approach allowed most reliable 11β-HSD1 suppression and a clear delineation of response patterns. Inactive DHC, which requires conversion to corticosterone to trigger glucocorticoid responses, induced GILZ, myostatin and Fbxo32 expression and suppressed TNFα-stimulated IL6 expression in 11β-HSD1 expressing myotubes, but not in myotubes with genetic deletion of 11β-HSD1. Reduced DHC responses for myostatin and arguably GILZ in the presence of TNF could indicate possible glucocorticoid resistance, a finding that would require further characterization [25].

There remains uncertainty to what extent the observed muscle phenotype in vivo is caused by 11β-HSD1-mediated glucocorticoid activation directly within muscles, as opposed to 11β-HSD1 activity elsewhere in the body. Previous studies suggest that 11β-HSD1 activity in myeloid cells is important for immunosuppression by glucocorticoids in vivo [16], while 11β-HSD1 activity in liver appeared irrelevant for myopathic effects [8]. Variable activity of 11β-HSD1 in different tissue types may determine the balance of beneficial immunosuppressive versus harmful metabolic effects with glucocorticoid therapy. Further research with tissue-targeted glucocorticoid delivery or 11β-HSD1 suppression is needed to clarify this concept and advance the safety of glucocorticoid-based therapies.

Clinical data on the potential benefits of 11β-HSD1 inhibition on muscle metabolism is emerging. Observational studies have reported a correlation of 11β-HSD1 expression in muscle with total lean mass and muscle strength in healthy elderly [26,27]. Furthermore, a clinical trial found that the 11β-HSD1 inhibitor AZD4017 increased total lean mass in young overweight women [11]. Finally, the TICSI trial is of particular interest in relation to the findings reported here (ClinicalTrials.gov (accessed on 12 July 2021) ID: NCT03111810). It aims to determine whether co-administration of an 11β-HSD1 inhibitor can limit the metabolic side effects of prednisolone, with results expected to report in the near future. However, safety data will be needed to confirm that 11β-HSD1 inhibitors do not exacerbate inflammatory pathology before using them in patients requiring immunosuppressive therapy. Given that 11β-HSD1 mediates both immunosuppressive and metabolic effects of glucocorticoids, tissue-targeted strategies may hold greater promise than systemic 11β-HSD1 inhibition.

## 4. Materials and Methods

All biochemical reagents used are from Sigma, Dorset, United Kingdom, unless stated otherwise.

### 4.1. Human Skeletal Muscle Biopsies

Adult patients with hip osteoarthritis (OA) or rheumatoid arthritis (RA) consented to collection of quadriceps muscle biopsies (150–200 mg) during elective joint replacement surgery, following ethical approval (REC reference 14/ES/1044 & NRES 16/SS/0172). Current glucocorticoid therapy was an exclusion criterion. Fresh muscle tissue was used either immediately for enzymatic activity analysis, or snap-frozen in liquid nitrogen for later real-time PCR analysis.

### 4.2. Animal Models

Transgenic mice with chronic systemic overexpression of human *TNF-α* (TNF-tg mice) were used as a model for chronic inflammation and polyarthritis. TNF-tg animals were provided by Professor George Kollias (Biomedical Sciences Research Center ‘Alexander Fleming’, Athens, Greece). This model, first described by Keffer et al. [28], relies on replacement of the 3′-untranslated region of the human TNFα gene with the 3′-untranslated region of the β-globin gene, which greatly increases transcriptional efficiency, stability and expression. Human TNFα has homology with murine TNFα and effectively binds murine TNF-R1 receptors, but not murine TNF-R2 receptors, resulting in less severe inflammatory manifestations than chronic overexpression of murine TNFα [29,30]. Transgene expression affects multiple tissues (joints, brain, kidney, spleen, thymus) with confirmed expression in murine skeletal muscle tissue [13,28]. We have previously demonstrated high levels of human TNFα transgene expression and equivalent levels of native murine TNFα expression in skeletal muscle of TNF-tg mice compared to wild-type mice [13]. The characteristic phenotype of these animals involves progressive polyarthritis from 6 weeks of age. The inflammatory and musculoskeletal phenotype of these TNF-tg mice has previously been described [13,15,16,31]. TNF-tg mice were crossed with 11β-HSD1 global KO mice (11βKO) as previously described to generate TNF-tg^11βKO^ animals [16]. All animals were maintained on C57BL/6 background and littermates with intact 11β-HSD1 gene served as respective controls. Preserved human TNFα transgene expression in skeletal muscle tissue of double mutant mice has previously been validated [13] and was confirmed for this study (Supplementary Figure S1).

Male mice were housed in standard conditions with ad lib access to standard chow and water. Animals were scored twice weekly for clinical parameters of inflammation using a validated template [13,31] from 28 days of age until the end of the experiment. Treatment with glucocorticoids or vehicle started from 32 days of age, coinciding with onset of measurable polyarthritis, and continued for 3 weeks. Drinking water was supplemented with either corticosterone (100 μg/mL, 0.66% ethanol), or vehicle (0.66% ethanol), with an average consumption of 1.25 mg per day. Mice were sacrificed at the end of 3 weeks’ treatment. Adrenal glands, tibialis anterior (TA) and quadriceps muscle were dissected, weighed and weights normalized to total body weights. Muscles were snap frozen in liquid nitrogen and stored at −80 °C for later biochemical analysis. Experiments complied with the Animal (Scientific Procedures) Act 1986 and received approval from the Birmingham Ethical Review Subcommittee (project license P51102987).

### 4.3. 11β-HSD1 Enzymatic Activity Assay

Confluent cells or fresh tissues were incubated in medium containing 100 nmol/L cortisone (for human samples) or 100 nmol/L DHC (for rodent samples) along with tracer amounts of tritiated cortisone or tritiated DHC (Perkin Elmer, Beaconsfield, UK). Steroids were extracted in dichloromethane and separated by thin-layer chromatography with ethanol/chloroform (8:92) as the mobile phase. Thin-layer chromatography plates were analyzed with a Bioscan imager (Bioscan, Washington, DC, USA), and the fractional conversion of steroids was calculated. The protein concentration was determined with the Bio-Rad Protein assay using the Bradford method (Bio-Rad, Hercules, CA, USA). Experiments were performed in triplicate, and enzymatic activity is reported as pmol product per mg protein per hour.

### 4.4. Primary Murine Muscle Cell Culture

Murine TA muscles were used to generate primary cultures of differentiated myotubes as previously described [32,33]. TA muscles were removed from WT and 11βKO mice at 9 weeks and digested in type 1 collagenase at 37 °C for 2 h, before isolating individual fibers. Fibers were plated in satellite media (DMEM High Glucose, 30% FBS, 10% HS, 1% Chick Embryo Extract, 10 ng/mL basic fibroblast growth factor) and grown in Matrigel™-coated plates (Corning Life Sciences, Flintshire, UK). Satellite cells migrating from muscle fibers were removed and cultured in proliferation medium (DMEM High Glucose, 10% HS, 0.5% Chick Embryo Extract) until confluent. Primary myoblasts were then grown in differentiation medium (DMEM High Glucose, 2% HS) for five days until syncytialized myotubes formed. To investigate effects of inflammation and glucocorticoids, differentiated myotubes were incubated in media with added TNF-α (10 ng/mL) and/or dehydrocorticosterone (1 µmol/l) for 24 h.

### 4.5. RNA Isolation and Analysis of Gene Expression

RNA was extracted by mechanical suspension and lysis of muscle in TRIzol™ reagent (Thermo Fisher Scientific, Loughborough, UK). Phase separation and RNA precipitation was performed with the addition of chloroform and 2-propanol. RNA precipitates were reconstituted in RNase-free water and stored at −80 °C. cDNA was generated by reverse transcription in accordance with the manufacturer’s protocol (Multiscribe™, Thermo Fisher Scientific, Loughborough, UK). Expression of specific genes was assessed by real-time PCR using TaqMan^®^ Gene Expression Assays (Thermo Fisher Scientific, Loughborough, UK) on an ABI7500 system (Applied Biosystems, Warrington, UK). Final reactions contained 2X TaqMan PCR Mastermix (Thermo Fisher Scientific, Loughborough, UK), 200 nmol TaqMan probe and 25–50 ng cDNA. The abundance of specific mRNAs in a sample was normalized to the housekeeper gene GAPDH by calculating ΔCt values (Ct target − Ct GAPDH). For graphical illustrations, standardized expression values were transformed with the formula (2^−ΔCt^) × 1000. For describing changes between experimental and control groups, the difference of ΔCt values as calculated as ΔΔCt = ΔCt[experimental group] − ΔCt[control group] and reported as fold change = 2^ΔΔCt^.

### 4.6. Histological Analysis of Muscle

Murine quadriceps muscles were embedded in paraffin and cut to 10 µm sections for histology. Samples were stained with hematoxylin and eosin prior to quantitative analysis of fiber size distribution using Image J software [34]. Measurements were taken in three 200 µm^2^ regions of the vastus medialis for six mice per group.

### 4.7. Western Blots

Protein content of quadriceps muscles was homogenized in an ice-cold lysis buffer (50 mM Tris, 1 mM EDTA, 10 mM Na-B-Glycerophosphate, 5 mM Na-Pyrophosphate, 1 mM Benzamidine, 250 mM Sucrose, 50 mM NaF, 0.1% β-Mercaptoethanol, 1 mM Na2VO3, 1% Triton X and Protease Inhibitor Cocktail (Merck Life Science, Dorset, UK)). Proteins were denatured in Laemmli buffer at 95 °C for 5 min. 20 µg of proteins were resolved in Tris-glycine SDS-PAGE gels and transferred onto polyvinylidene difluoride membranes. After blocking, membranes were probed with the primary antibody overnight at 4 °C. All primary antibodies were obtained from Cell Signaling Technology, Inc. and diluted at 1:1000 in TBS-Tween plus 5% BSA or skimmed milk (p-FOXO1 #9464, FOXO1 #2880, p-S6 Ribosomal Protein (Ser235/236) #4856, S6 Ribosomal Protein #2217). Signal detection used horseradish peroxidase-conjugated secondary antibodies and ECL substrate. The membrane was stained with Ponceau S solution (0.2% Ponceau S in 1% acetic acid; Sigma-Aldrich Chemie) to control for protein loading.

### 4.8. Statistical Analysis

Data was analyzed by unpaired *t*-test, one way and two-way ANOVA with Tukey post-hoc analysis or Pearson correlation analysis, or non-parametric equivalent tests as appropriate using GraphPad Prism [35]. Aggregate data are reported as mean ± standard error, unless otherwise specified. Statistical significance was defined as *p*-value < 0.05 (* *p* < 0.05; ** *p* < 0.01; *** *p* < 0.001; no asterisk or NS *p* > 0.05).

## Figures and Tables

**Table 1:** Patients’ characteristics for human skeletal muscle biopsies.

Patient Details	Patients with OA (n = 12)	Patients with RA (n = 10)	Group Comparison (*p*-Value)
Age (years)	66.2 + 3.3	65.7 + 4.1	0.93
CRP (mg/L)	2.3 + 1.7	11.0 + 3.5	0.03
ESR (mm/h)	1.9 + 1.0	24.13 + 7.1	0.001
Methotrexate (n)	0	5	na
Anti-TNF therapy (n)	0	2	na
Prednisolone	0	0	na

CRP, C-reactive protein; ESR, erythrocyte sedimentation rate.

## Data Availability

Data and materials within this manuscript can be made available upon reasonable request to the corresponding author.

## Supplementary Materials

The following are available online at https://www.mdpi.com/article/10.3390/ijms22157828/s1.

## Abbreviations

11β-HSD1	11beta-hydroxysteroid dehydrogenase type 1
DHC	dehydrocorticosterone
HPA	hypothalamic-pituitary-adrenal
OA	osteoarthritis
RA	rheumatoid arthritis
TNF-tg	transgenic expression of human TNF-α
11βKO	global genetic deletion of 11β-HSD1
WT	wild-type genotype without genetic modifications
TA	tibialis anterior
NS	not significant
ND	not detectable
ESR	erythrocyte sedimentation rate
CRP	C-reactive protein
