# Supplementary material for: Global Deletion of 11β-HSD1 Prevents Muscle Wasting Associated with Glucocorticoid Therapy in Polyarthritis
Source: Int J Mol Sci. 2021 Jul 22;22(15):7828. doi: 10.3390/ijms22157828 (PMC8346140; doi:10.3390/ijms22157828)
Supplement: Supplementary file 1 [file ijms-22-07828-s001.zip › ijms-1245636-supplementary.pdf]

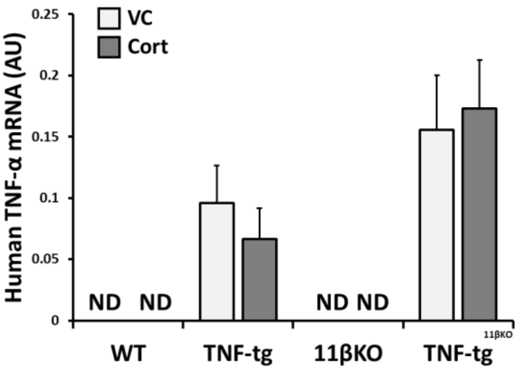

Supplementary Figure 1. Gene expression (AU) of human TNFα transgene expression determined by RT PCR in muscle homogenates from wild-type (WT), human TNFα transgenic (TNF-tg), 11β-HSD1 global knock-out (11βKO) and double mutant TNF-tg$^{11\beta KO}$ animals receiving either vehicle or corticosterone (100 μg/mL) in the drinking water for 3 weeks. Values are expressed as mean±SE, n=6 per group. No statistically significant differences were detected for TNF-tg and TNF-tg$^{11\beta KO}$ groups using two-way analysis of variance with Tukey post hoc analysis. WT = Wild type, 11βKO = 11β-HSD1 genetic deletion, VC = Vehicle Control, Cort = Corticosterone, ND = Non-detectable.

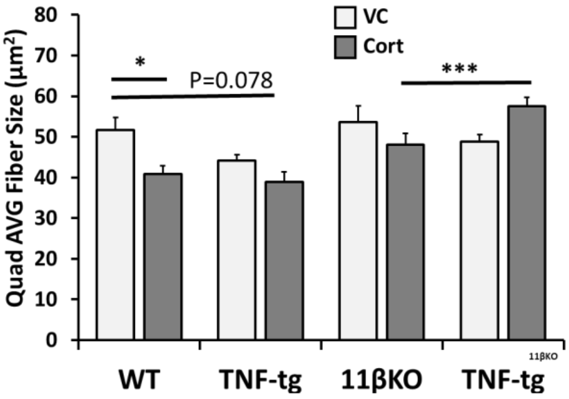

Supplementary Figure 2: Average quadriceps muscle fibre cross sectional area (μm^2) in wild-type (WT), human TNFα transgenic (TNF-tg), 11β-HSD1 global knock-out (11βKO) and double mutant TNF-tg$^{11\beta KO}$ mice receiving either vehicle or corticosterone (100 μg/mL) in the drinking water for 3 weeks. Values are expressed as mean±SE, n=6 per group. WT = Wild type, 11βKO = 11β-HSD1 genetic deletion, VC = Vehicle Control, Cort = Corticosterone.
